# Supplementary material for: Unlocking the potentials of cyanobacterial photosynthesis for directly converting carbon dioxide into glucose
Source: Nat Commun. 2023 Jun 9;14:3425. doi: 10.1038/s41467-023-39222-w (PMC10256809; doi:10.1038/s41467-023-39222-w)
Supplement: Supplementary file 1 — Supplementary Information [file 41467_2023_39222_MOESM1_ESM.pdf]

Unlocking the potentials of cyanobacterial photosynthesis for directly converting carbon dioxide into glucose

Zhang and Sun *et al.*

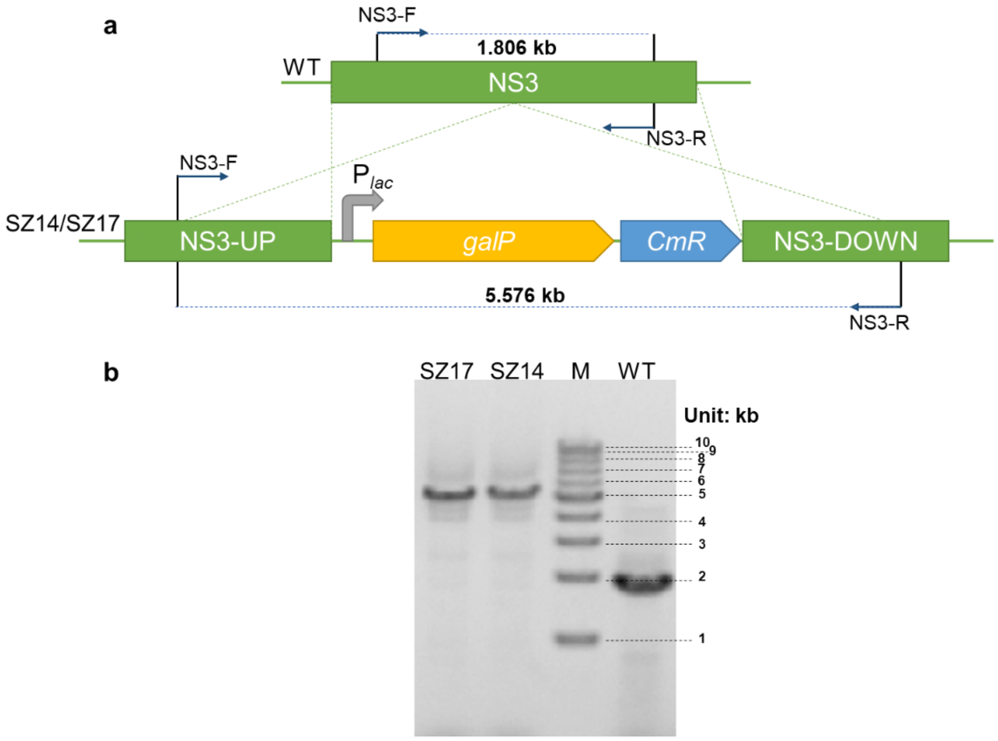

Supplementary Figure 1. Introduction of the glucose transporter GalP in *Synechococcus elongatus* PCC 7942. (a) Construction strategy of GalP expression system controlled by P_{lac} in PCC 7942. (b) Genotype identification of the SZ14, SZ17, and WT by PCR (expected size of 5.576 kb, 5.576 kb, and 1.806 kb, respectively). Each experiment was replicated more than twice to ensure their reliabilities. P_{lac}, isopropyl-D-1-thiogalactopyranoside (IPTG)-inducible promoter. NS3, neutral site 3 of PCC 7942.

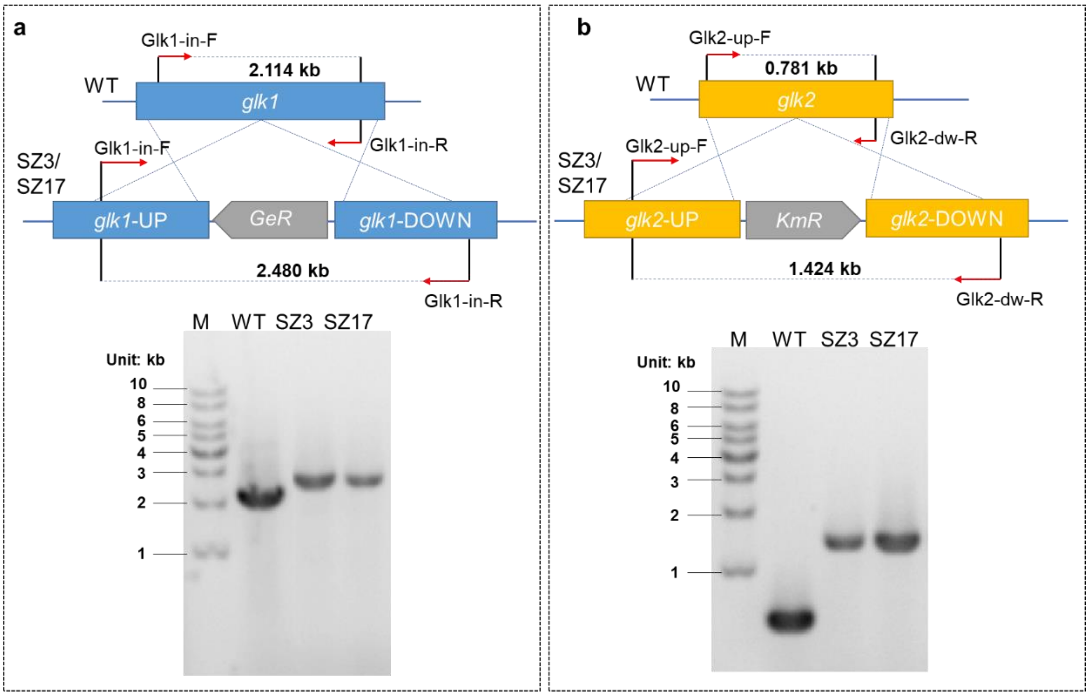

Supplementary Figure 2. Knockout of glucose phosphorylation system of PCC 7942-WT and PCC 7942-SZ14 strains. (a) The strategy of knocking out the *glk1 (synpcc7942_0221)* gene and the genotype verification of engineered strains (expected sizes of 2.480 kb and 2.114 kb, respectively). (b) The strategy of knocking out the *glk2 (synpcc7942_2111)* gene and the genotype verification of engineered strains (expected sizes of 1.424 kb and 0.781 kb, respectively). Each experiment was replicated more than twice to ensure their reliabilities.

a

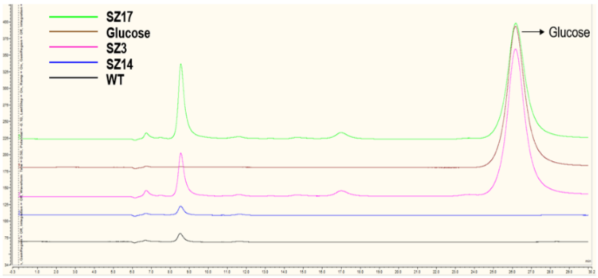

b

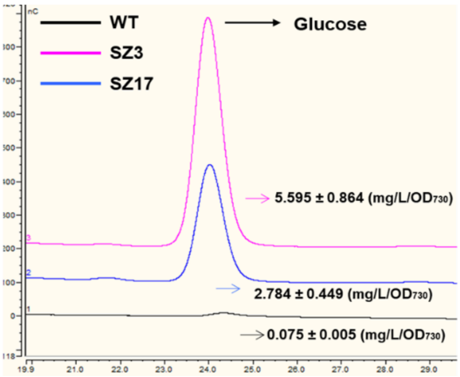

Supplementary Figure 3. Glucose production of *Synechococcus* strains with or without *glk* deletion. (a) WT, SZ3 (WT-Δ*glk1*-Δ*glk2)*, SZ14 (Δ*NS3::galP*), and SZ17 (WT-Δ*glk1*-Δ*glk2*-Δ*NS3::galP*) cells were cultured to the 16th day under standard cultivation conditions (BG11 medium, 200 μmol photons/m^2/s, 3% CO_2 bubbling for carbon supply) were centrifuged at 12000 g for 1 min. Then the supernatants were filtered through the 0.22 μm polyethersulfone membranes into clean vials and directedly analyzed using an ICS5000+ (DIONEX, Thermo Scientific, USA) ion-exchange chromatography system equipped with an electrochemical detector and a Dionex CarboPac MA1 analytical column (4×250 mm, Thermo Scientific, Waltham, MA, USA). The column was equilibrated and eluted with 480 mM NaOH at a flow rate of 0.4 mL/min. The glucose standard sample was used as a control. (b) WT, SZ3, and SZ17 cells cultivated to the 6th day under standard cultivation conditions (BG11 medium, 200 μmol photons/m^2/s, 3% CO_2 bubbling for carbon supply) were centrifuged and the cells pellets were treated with 1 mL 80% ethanol (volume to volume) at 65°C for 4 h for extractions. After centrifugation at 12,000 g for 5 min, the supernatant was transferred to a clean tube and dried under a stream of N_2 at 55°C. Subsequently, the dry residues were dissolved in ultrapure water, and the dissolved solution was filtered into clean vials with a 0.22 μm filtration membrane and assayed as the described above. Data are presented as mean values ± SD (n=4 biological replicates) in b. Source data are provided as a Source Data file.

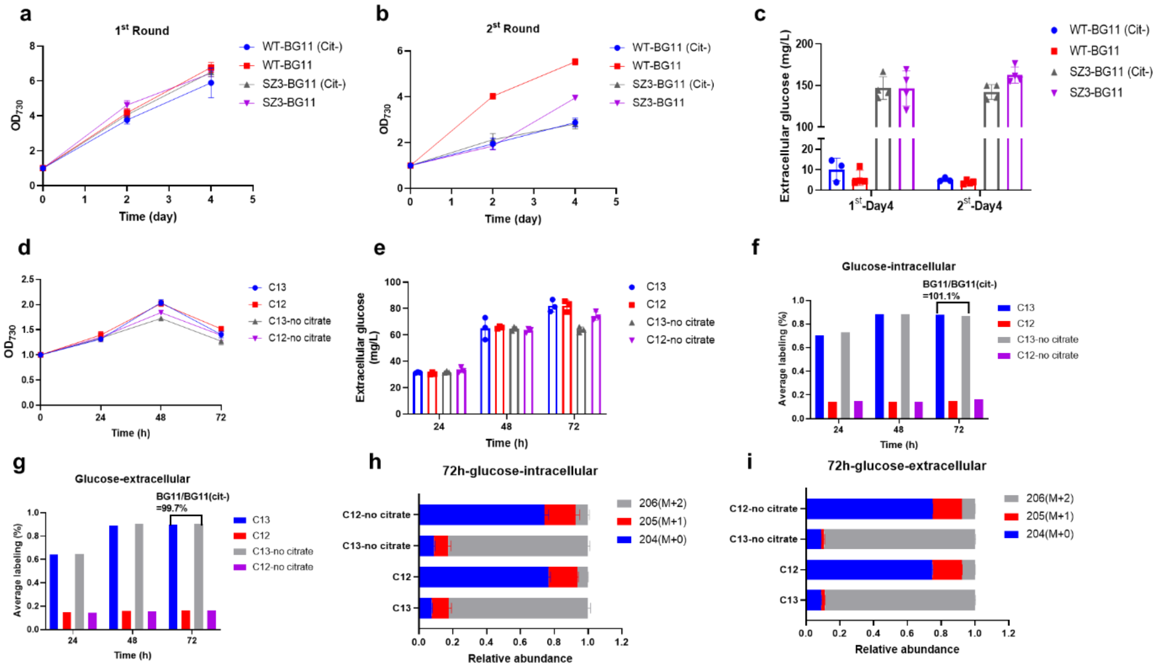

Supplementary Figure 4. Effects of the organic carbon contents in BG11 culture medium on cell growth and glucose production of SZ3. (a) & (b) Two rounds cultivation of WT and SZ3 cells in BG11 culture medium in column photobioreactors with or without citrate and ferric amine citrate contents, termed as BG11 and BG11 (Cit-), respectively. (c) Glucose production of WT and SZ3 cultivated in BG11 and BG11 (Cit-) medium in column photobioreactors. (d) & (e) Cell growth and glucose production of SZ3 cells in shake flasks in BG11 and BG11 (Cit-) culture medium with $NaH^{13}CO_3$ or $NaH^{12}CO_3$ as the carbon source. (f) & (g). Average ^{13}C isotope incorporating rates of synthesized intracellular (f) and extracellular (g) glucose of SZ3 cells cultivated in BG11 and BG11 (Cit-) with $NaH^{13}CO_3$ or $NaH^{12}CO_3$ as the carbon source. (h) & (i) Relative abundance of the SZ3 synthesized intracellular (h) and extracellular glucose (i) mass isotopomer with $NaH^{13}CO_3$ or $NaH^{12}CO_3$ as the carbon source after 72 h cultivation. Data are presented as mean values ± SD. All sample size n (biological replicates) in a, b, c is 4 except that n of the WT-BG11 (Cit-) column in c is 3. All sample size n (biological replicates) in d, e, h, i is 3. Source data are provided as a Source Data file.

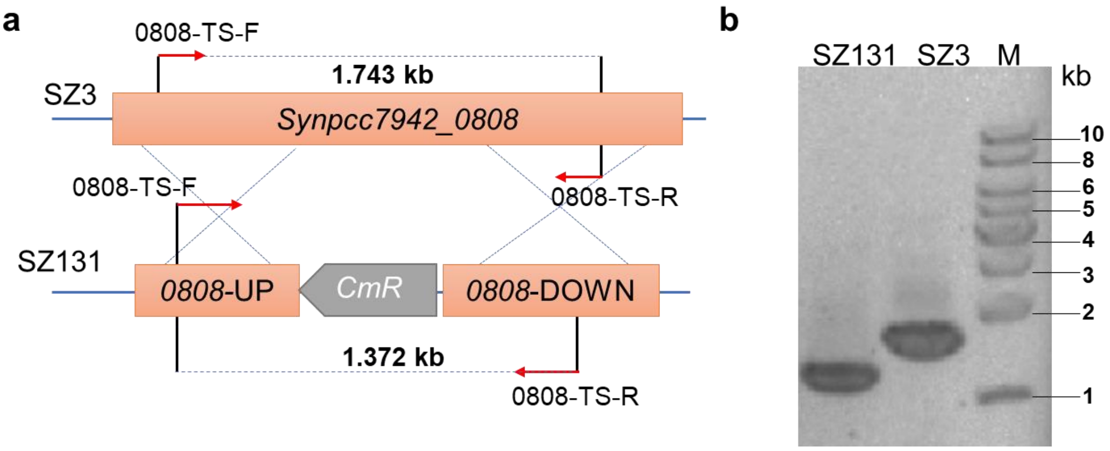

Supplementary Figure 5. The knockout of sucrose phosphate synthase encoding gene in SZ3 strain. (a) Construction strategy of *sps*-deficient strain. (b) Genotype identification of the SZ131 and SZ3 by PCR (expected sizes of 1.372 kb and 1.743 kb, respectively). Each experiment was replicated more than twice to ensure their reliabilities.

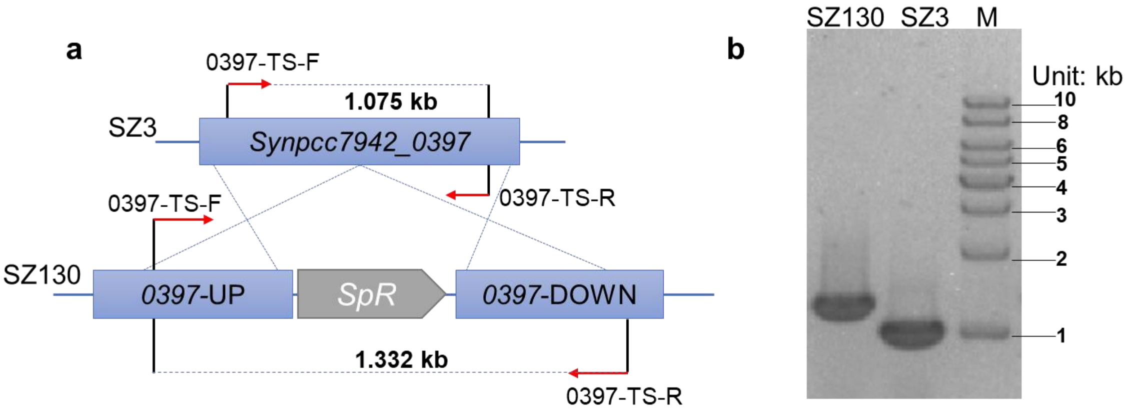

Supplementary Figure 6. The knockout of invertase encoding gene in SZ3 strain. (a) Construction strategy of *invA*-deficient strain. (b) Genotype identification of the SZ130 and SZ3 by PCR (expected sizes of 1.332 kb and 1.075 kb, respectively). Each experiment was replicated more than twice to ensure their reliabilities.

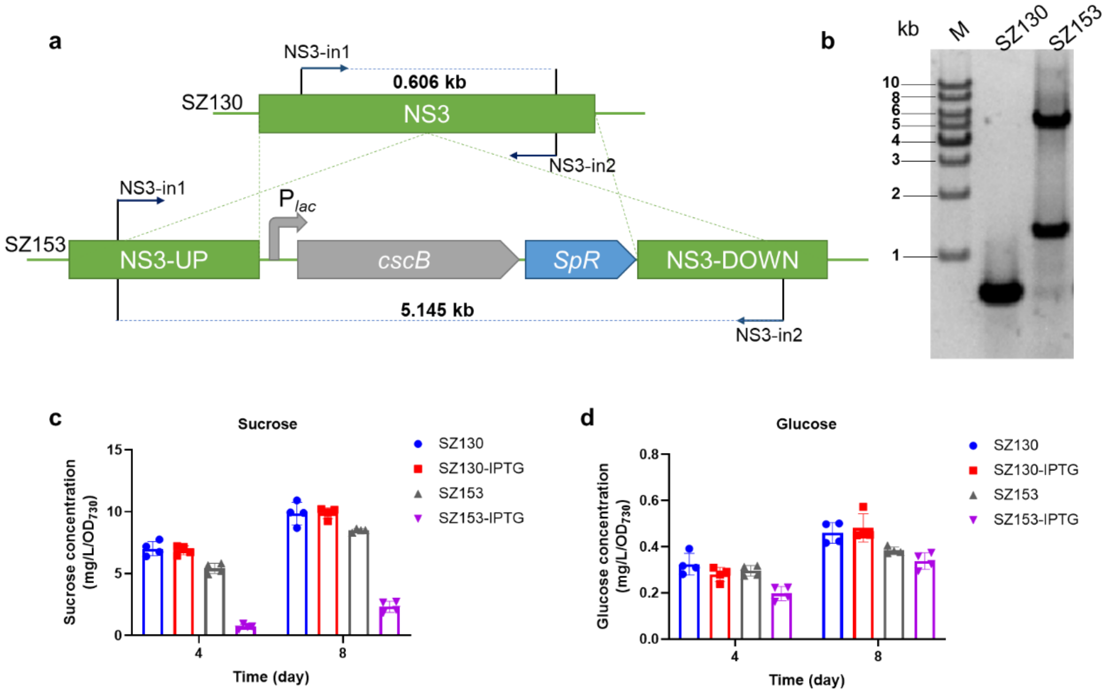

Supplementary Figure 7. The introduction of the sucrose transporter gene *cscB* in SZ130 strain. (a) Construction strategy of *cscB*-expressing strain. (b) Genotype identification of the SZ153 and SZ130 by PCR (expected sizes of 5.145 kb and 0.606 kb, respectively). Each experiment was replicated more than twice to ensure their reliabilities. P_{lac}, isopropyl-D-1-thiogalactopyranoside (IPTG)-inducible promoter. (c) Intracellular sucrose concentration of SZ130 and SZ153 with or without IPTG induction (for CscB expression). (d) Intracellular glucose concentration of SZ130 and SZ153 with or without IPTG induction (for CscB expression). Data are presented as mean values ± SD. All sample size n (biological replicates) in c and d is 4. Source data are provided as a Source Data file.

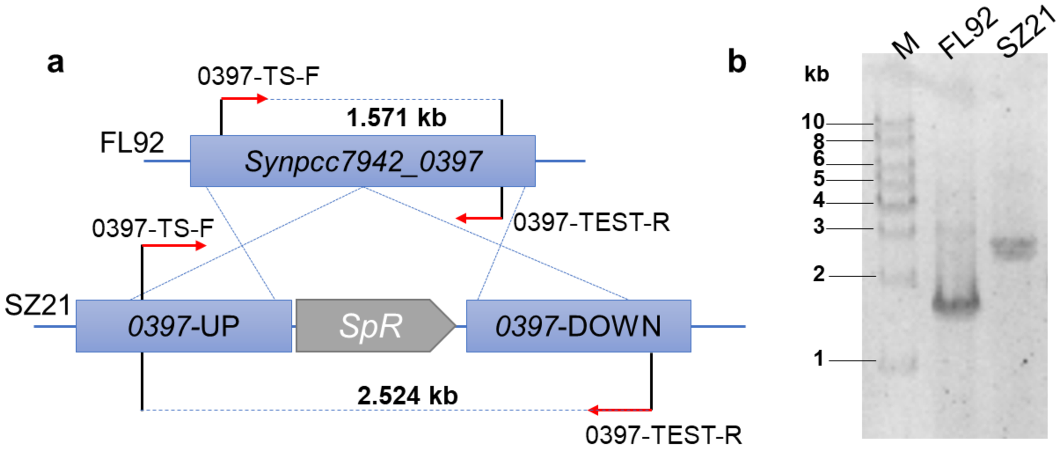

Supplementary Figure 8. The knockout of invertase encoding gene in FL92 strain. (a) Construction strategy of *invA*-deficient strain. (b) Genotype identification of the SZ21 and FL92 by PCR (expected sizes of 2.524 kb and 1.571 kb, respectively). Each experiment was replicated more than twice to ensure their reliabilities.

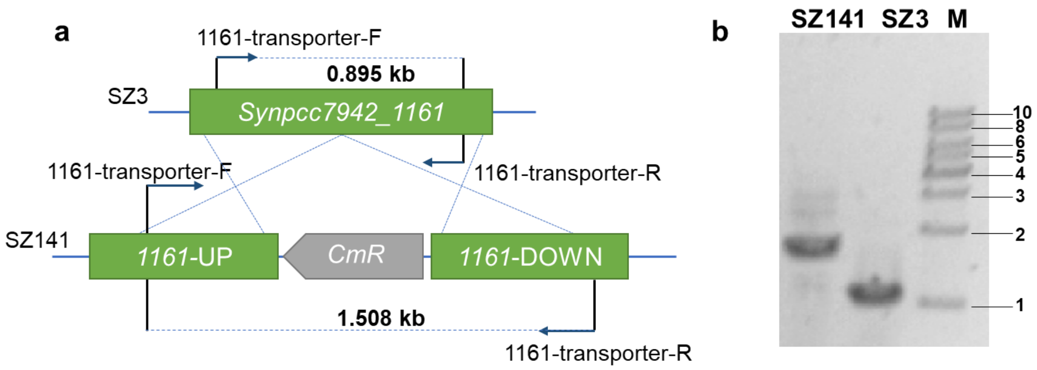

Supplementary Figure 9. The knockout of *synpcc7942_1161* in SZ3 strain. (a) Construction strategy of the *synpcc7942_1161*-deficient strain of SZ3. (b) Genotype identification of the SZ141 and SZ3 by PCR (expected sizes of 1.508 kb and 0.895 kb, respectively). Each experiment was replicated more than twice to ensure their reliabilities.

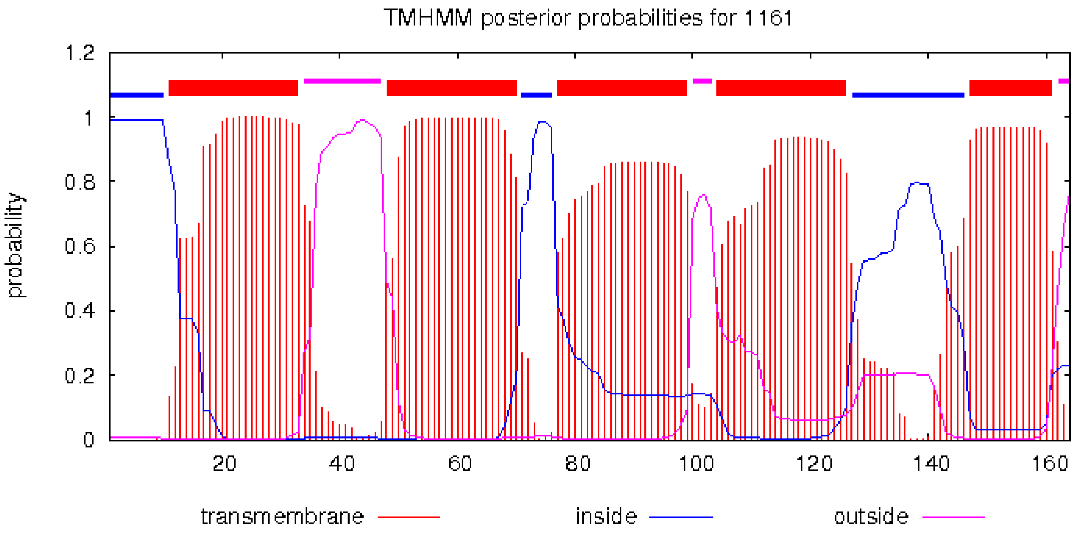

Supplementary Figure 10. Prediction of the transmembrane domains of the divalent metal transporter encoded by *synpcc7942_1161*. Figure was generated by the online prediction server: http://www.cbs.dtu.dk/services/TMHMM/

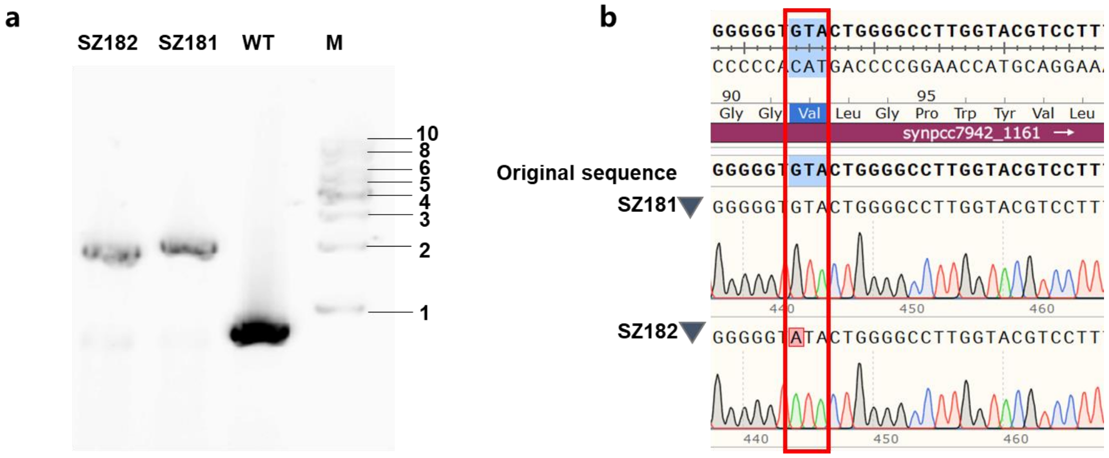

Supplementary Figure 11. Introduction of the *synpcc7942_1161*-G274A point mutation in PCC 7942. (a) Genotype identification of the SZ181 and SZ182 by PCR. Primers 1161-regulator-F and 1161-regulator-R were used for PCR identification (expected sizes of 1.867 kb, 1.867 kb, and 0.869 kb, respectively). Each experiment was replicated more than twice to ensure their reliabilities. (b) Sanger DNA sequencing of the amplified *synpcc7942_1161* gene regions of mutants SZ181 and SZ182.

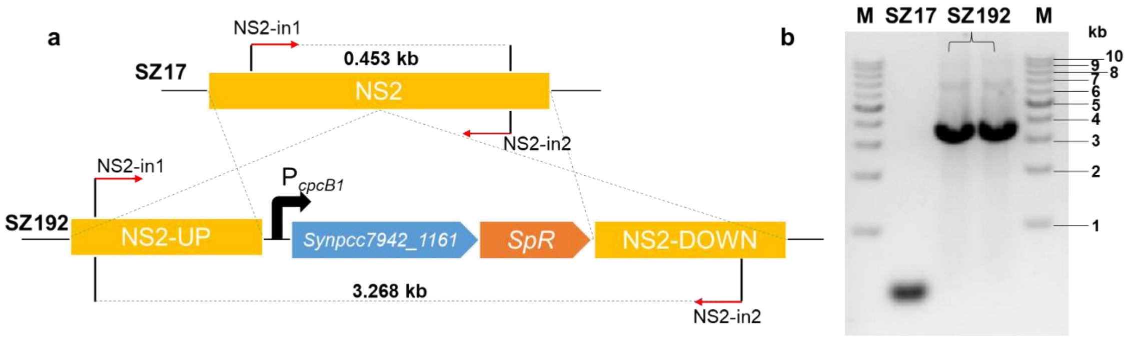

Supplementary Figure 12. Overexpression of the *synpcc7942_1161* gene in SZ17 strain. (a) Construction strategy of *synpcc7942_1161*-overexpressing strain. (b) Genotype identification of the SZ192 and SZ17 by PCR (expected sizes of 3.268 kb and 0.453 kb, respectively). Each experiment was replicated more than twice to ensure their reliabilities.

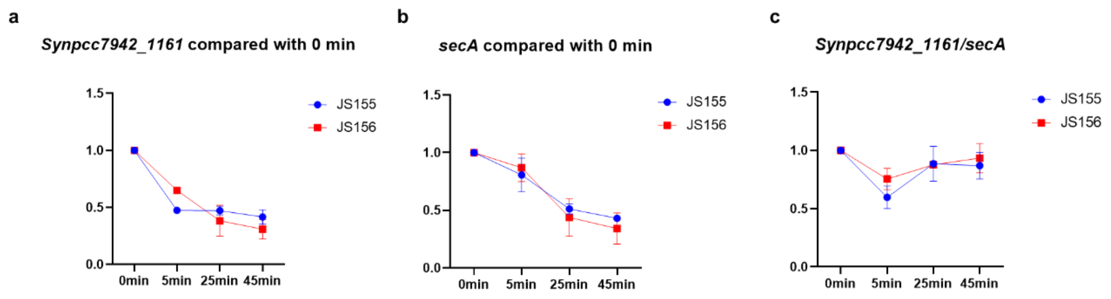

Supplementary Figure 13. ***Synpcc7942_1161*** **mRNA stability comparation in JS155 and JS156.** (a) Relative *Synpcc7942_1161* mRNA levels in cells of JS155 and JS156 were measured by quantitative RT–PCR normalized to that at 0 min (before ActD was added). (b) Relative *secA* mRNA levels were measured by quantitative RT–PCR normalized to that at 0 min (before ActD was added). (c) Relative folds of expression were calculated by normalizing mRNA level to that at 0 min (before ActD was added) for *secA* (internal reference) and *Synpcc7942_1161*-overexpressed groups. Data are presented as mean values ± SD (n=3 biological replicates). Source data are provided as a Source Data file.

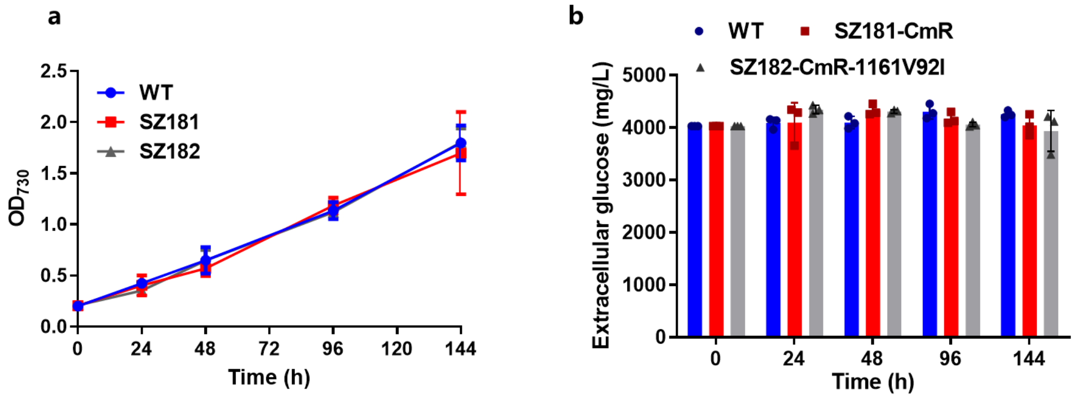

Supplementary Figure 14. Effects of the *synpcc7942_1161*-G274A point mutation on glucose transport capacity of PCC 7942. (a) Growth profiles of WT, SZ181, and SZ182. (b) Extracellular glucose production of WT, SZ181, and SZ182. 4 g/L glucose was added to the initial BG11 medium. Data are presented as mean values ± SD (n=3 biological replicates). Source data are provided as a Source Data file.

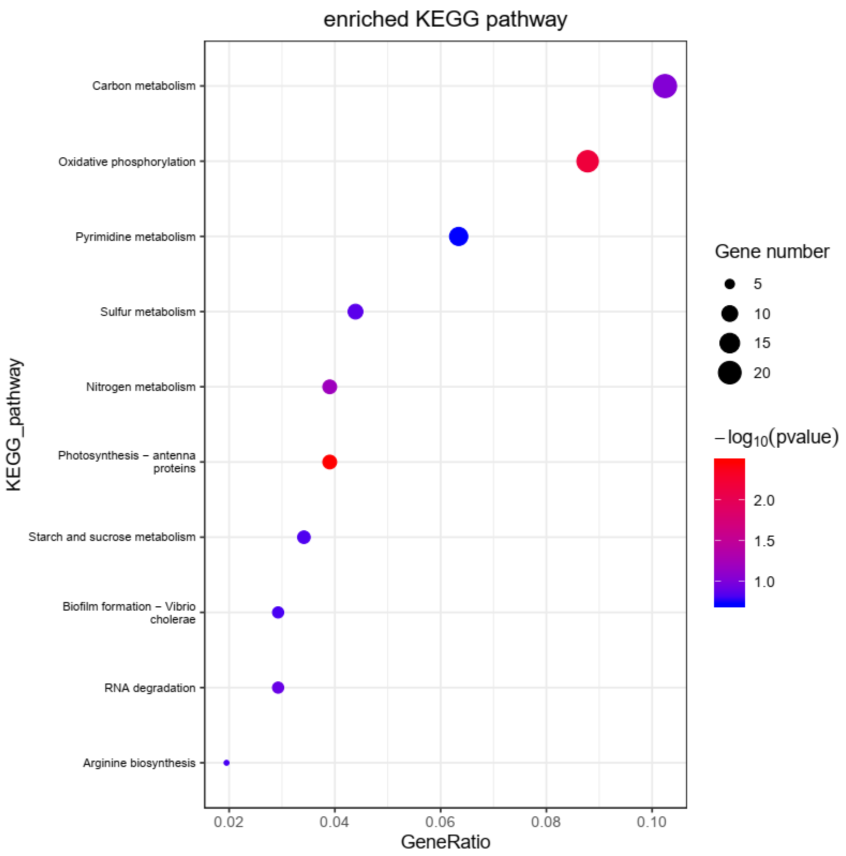

Supplementary Figure 15. KEGG pathway enrichment analysis of the transcriptome differences between SZ3 and WT. Shown were up-regulated and down-regulated KEGG pathways between SZ3 and WT. The dot size indicates the number of genes differentially expressed in the certain pathway. Groups were compared with Fisher's exact test for outcome data and the $-\log_{10}(p\text{-value})$ was shown with colors. Source data are provided as a Source Data file.

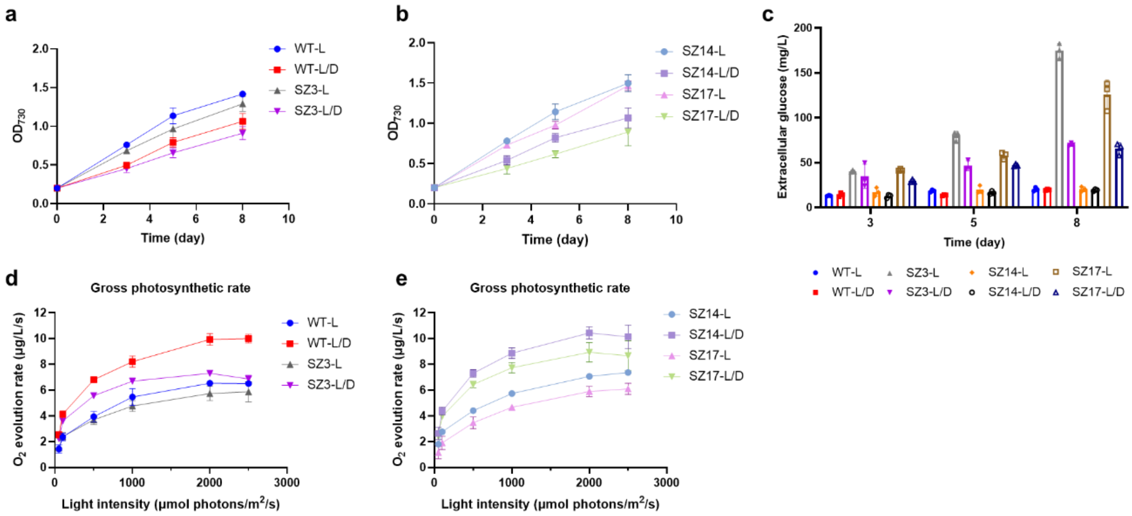

Supplementary Figure 16. Influence of glucokinase deficiency on cell growth of PCC 7942 cultivated under continual light conditions or Light-Dark cycle conditions. (a) & (b) The two recombinant strains with removed glucokinase activities (SZ3 and SZ17) don't show significant response difference when cultivation mode was changed from continuous (24 h) light condition to L/D cycle (12h-12h light-dark) condition comparing with their respective controls (WT and SZ14). (c) Glucose secretions of the SZ3 and SZ17 were significantly decreased. In fact, the total carbon fixation for all the strains were decreased in the L/D cycle comparing with that in continuous light mode. (d) & (e) When cultivated in continuous light condition, whole chain oxygen evolution rates of the two GLK-deficient strains (SZ3 and SZ17) were lower than those of the respective controls (WT and SZ14). When cultivated in the L/D cycle mode, the difference between SZ3 and its control WT got even more obvious. Data are presented as mean values ± SD. All sample size n (biological replicates) in a and b is 4. All sample size n (biological replicates) in c, d and e is 3. Source data are provided as a Source Data file.

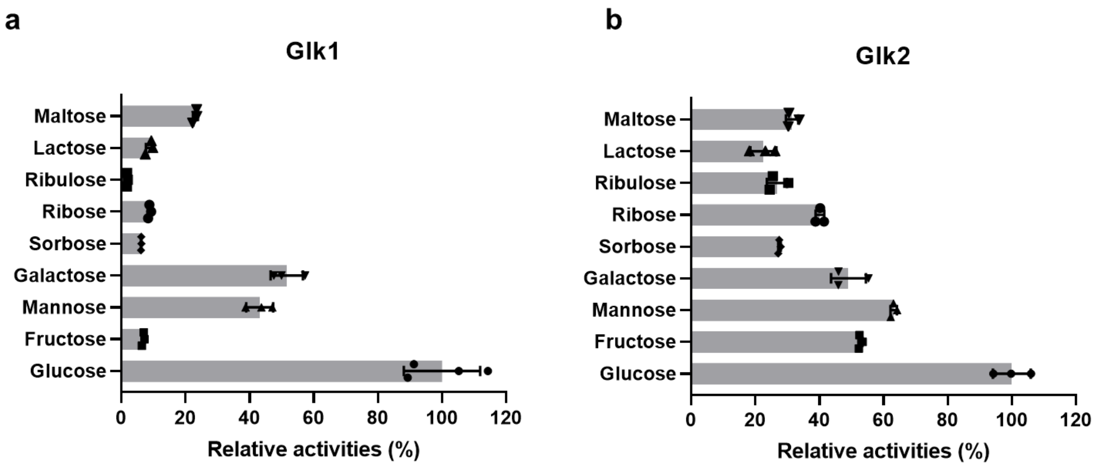

Supplementary Figure 17. Activities of Glk1 and Glk2 from PCC 7942 on catalyzing the phosphorylation process of different sugars. Specific activities of the two glucokinases (a, Glk1. b, Glk2) on each sugar would be compared to the activities on glucose to get the relative ratios. Data are presented as mean values ± SD. All sample size n (biological replicates) in a and b is 3 except that n of the Glucose column in a is 4. Source data are provided as a Source Data file.

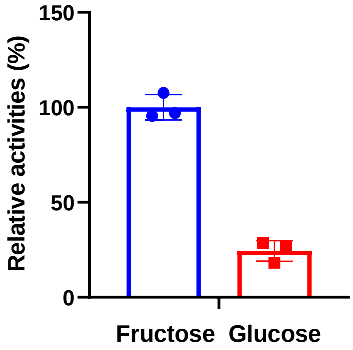

Supplementary Figure 18. Activities of fructokinase from PCC 7942 for phosphorylating fructose and glucose. Data are presented as mean values ± SD (n=3 biological replicates). Source data are provided as a Source Data file.

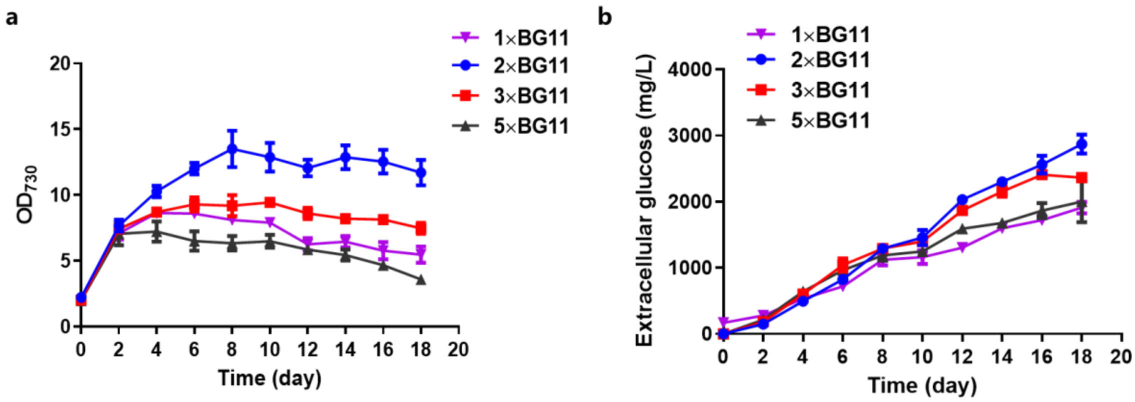

Supplementary Figure 19. Effects of BG11 media optimization on the SZ123 strain. Growth profiles (a) and extracellular glucose production (b) of SZ123 strain in different BG11 media. Data are presented as mean values ± SD (n=3 biological replicates). Source data are provided as a Source Data file.

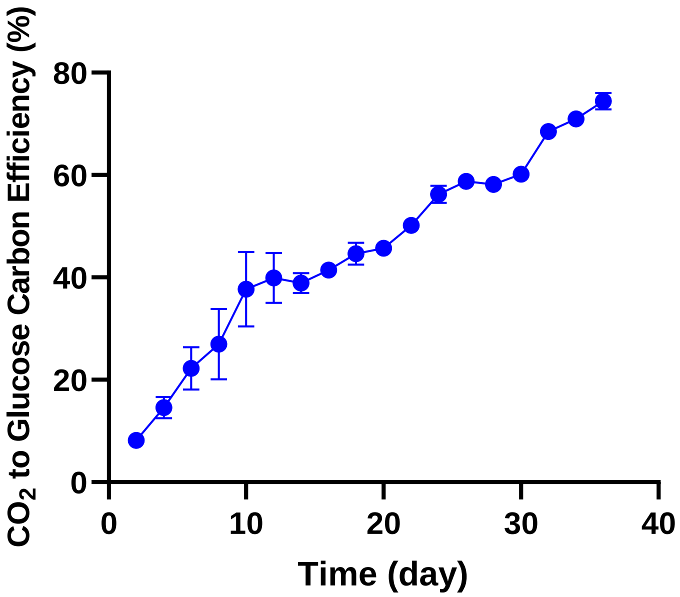

Supplementary Figure 20. Partitioning ratio of secreted glucose in the total biomass. During the sub-continual cultivations (3 batches), the glucose amounts and dry cellular weight (calculated with the OD_{730} using the equation "0.34 g/L/OD_{730}") were summed to obtain the total biomass, and the partition ratio of glucose in it would be calculated (as the carbon efficiency). Data are presented as mean values ± SD (n=3 biological replicates). Source data are provided as a Source Data file.

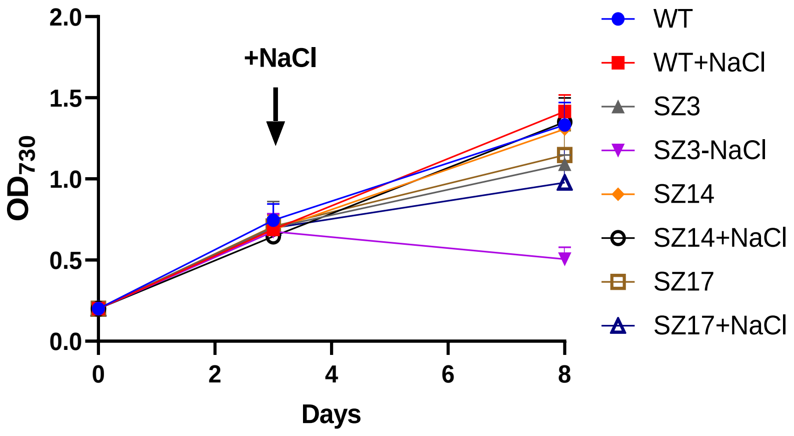

Supplementary Figure 21. Influence of glucokinase deficiency on salt-stress tolerances of PCC 7942 cells. 150 mM NaCl would be supplemented at Day 3 of the cultivation process and the cell densities would be calculated between the *Synechococcus* strains with or without glucokinases and GalP transporter. Data are presented as mean values ± SD (n=4 biological replicates). Source data are provided as a Source Data file.

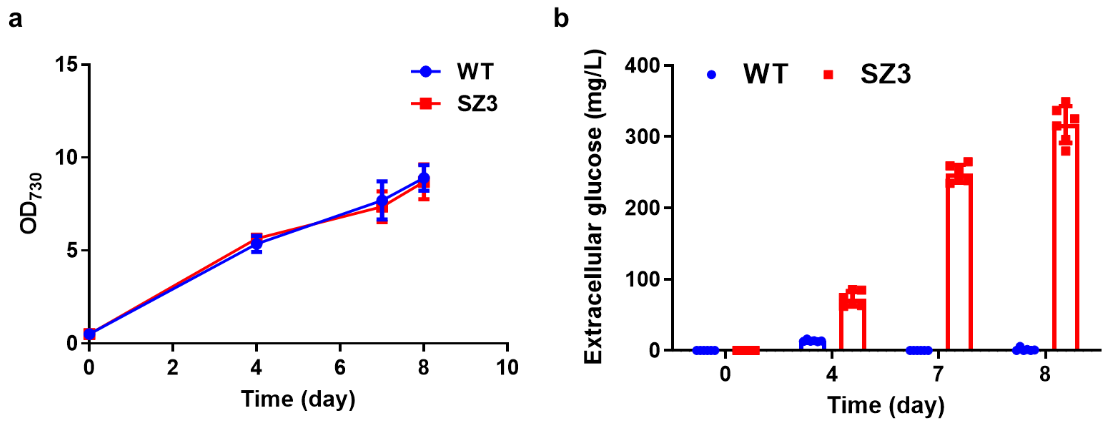

Supplementary Figure 22. The growths and glucose productions of PCC 7942 and SZ3 using for nontargeted UHPL-Q-TOF-MS-based metabolomics. Growth profiles (a) and extracellular glucose production (b) of WT and SZ3 strains. Data are presented as mean values ± SD (n=6 biological replicates). Source data are provided as a Source Data file.

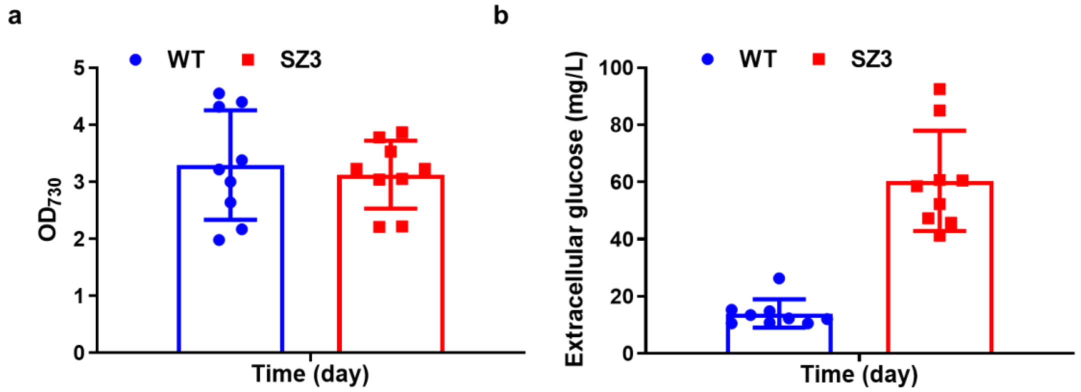

Supplementary Figure 23. The growths and glucose productions of PCC 7942 and SZ3 utilized for transcriptome analysis. OD_{730} (a) and extracellular glucose production (b) of WT and SZ3 cultivated to the 4th day. Data are presented as mean values ± SD (n=9 biological replicates). Source data are provided as a Source Data file.

Supplementary Table 1. Mutations (SNP and DEL) of SZ3 and SZ17 strains

**SZ3**				
**Definition**	**Gene symbol**	**Locus tag**	**NT mutation**	**AA mutation**
two-component system, OmpR family, response regulator RpaA	*Synpcc7942_0095*	*SYNPCC7942_RS00480*	A362G	Q121R
conserved hypothetical protein	*Synpcc7942_0863*	*SYNPCC7942_RS04450*	G70A	G24R
long-chain acyl-CoA synthetase	*Synpcc7942_0918*	*SYNPCC7942_RS04725*	T884C	L295P
bacterial/archaeal transporter family-2 protein	*Synpcc7942_1161*	*SYNPCC7942_RS05950*	G274A	V92I
transcriptional regulator, ArsR family	*Synpcc7942_1291*	*SYNPCC7942_RS06605*	G150A	R50R (synonymous)
CTP synthase	*Synpcc7942_1954*	*SYNPCC7942_RS09920*	T881C	V294A
transcriptional regulator, XRE family with cupin sensor domain	*Synpcc7942_2038*	*SYNPCC7942_RS10340*	C179T	A60V
conserved hypothetical protein	*Synpcc7942_2166*	*SYNPCC7942_RS11010*	G372T	X124Y (stopless)
conserved hypothetical protein	*Synpcc7942_1113*	*SYNPCC7942_RS05705*	NC_007604.1 (1130382..1131473, complement)	from 1129617 to 1130615, a 999 bp deletion
23S rRNA pseudouridine1911/1915/1917 synthase	*Synpcc7942_1112*	*SYNPCC7942_RS05700*	NC_007604.1 (1129444..1130385, complement)	
**SZ17**				
**Definition**	**Gene symbol**	**Locus tag**	**NT mutation**	**AA mutation**
two-component system, OmpR family, response regulator RpaA	*Synpcc7942_0095*	*SYNPCC7942_RS00480*	A362G	Q121R
conserved hypothetical protein	*Synpcc7942_0863*	*SYNPCC7942_RS04450*	G70A	G24R
long-chain acyl-CoA synthetase	*Synpcc7942_0918*	*SYNPCC7942_RS04725*	T884C	L295P
transcriptional regulator, ArsR family	*Synpcc7942_1291*	*SYNPCC7942_RS06605*	G150A	R50R (synonymous)
CTP synthase	*Synpcc7942_1954*	*SYNPCC7942_RS09920*	T881C	V294A
transcriptional regulator, XRE family with cupin sensor domain	*Synpcc7942_2038*	*SYNPCC7942_RS10340*	C179T	A60V
conserved hypothetical protein	*Synpcc7942_2166*	*SYNPCC7942_RS11010*	G372T	X124Y (stopless)
conserved hypothetical protein	*Synpcc7942_1113*	*SYNPCC7942_RS05705*	NC_007604.1 (1130382..1131473, complement)	from 1129617 to 1130615, a 999 bp deletion
23S rRNA pseudouridine1911/1915/1917 synthase	*Synpcc7942_1112*	*SYNPCC7942_RS05700*	NC_007604.1 (1129444..1130385, complement)	

Note: NT, nucleotide; AA, amino acid.

Supplementary Table 2. Increased intracellular carbohydrate metabolites of SZ3 strain

Metabolite	VIP	*p*-value	FC
3.alpha.-Mannobiose	2.7	5.10E-10	24.72
D-(+)-Melibiose	4.73	9.93E-09	128.15
D-Mannose*	5.16	2.66E-05	3.78
Isomaltose	2.79	1.81E-08	3.97
Maltopentaose	4.89	5.01E-09	5.09
Maltotetraose	1.28	2.07E-04	32.34
Maltotriose*	16.88	1.60E-08	4.02
N-Acetyl-D-glucosamine	1.75	5.19E-08	58.64
N-Acetyl-D-lactosamine	12.82	5.83E-07	1364.69
N-Acetylmannosamine	9.29	9.82E-09	253.49
UDP-D-Galactose	1.89	9.64E-04	2.32
D-Allose	6.37	1.26E-05	5.26
D-Fructose	4.02	4.80E-10	8.16
D-Lyxose	1.75	1.57E-06	3.95
D-Maltose	5.31	1.37E-08	19.89
D-Ribose	1.32	1.76E-05	4.09
D-Tagatose	3.02	8.94E-06	3.55
Galactinol	11.48	9.43E-08	484.37
L-Iditol	1.08	1.06E-04	6.57
Maltitol	6.42	5.70E-07	260.42
N-Acetylmannosamine	3.81	3.25E-08	365.01
N,N'-Diacetylchitobiose*	2.73	5.34E-05	28.36
Uridine diphosphate glucose	3.38	1.32E-04	2.93

Note: *indicates that the metabolites with differential abundances between SZ3 and WT are detected in both the positive ionization mode (ESI+) and the ionization negative mode (ESI-), the one with less change folds is shown here. VIP represents variable importance for the projection, the VIP score is based on the variable weight value obtained by the OPLS-DA model to measure the influence intensity and interpretation ability of the expression pattern of each metabolite on the classification and discrimination of each group of samples and assists the screening of metabolites. VIP score>1 is used as the screening criterion. The *p*-value is the statistical analysis value of the two-tailed unpaired Student's *t*-test statistics of the metabolites in the pairwise comparison group. *p*-value<0.05 represents a significant difference metabolite, and 0.05< *p*-value<1 represents a different metabolite. FC, fold change.

Supplementary Table 3. Decreased intracellular phosphorylated metabolites of SZ3 strain

Metabolite	VIP	*p*-value	FC
Adenosine 3',5'-diphosphate (PAP)	1.79	8.60E-03	0.44
Adenosine monophosphate (AMP)	6.67	5.00E-04	0.39
alpha-D-Glucose 1-phosphate	3.68	3.00E-06	0.12
Beta-D-Fructose 2-phosphate	1.74	1.00E-05	0.23
Cytidine 5'-diphosphate (CDP)	1.01	7.00E-11	0.09
Pyridoxamine 5'-phosphate	2.05	4.00E-05	0.01
D-Fructose-6-phosphate	1.48	5.00E-05	0.02
D-Glucose 6-phosphate	1.72	4.00E-05	0.12
2'-Deoxyguanosine 5'-monophosphate (dGMP)	4.4	2.30E-03	0.47
alpha-D-Galactose 1-phosphate	5.22	2.00E-05	0.11
D-Erythrose 4-phosphate	1.07	2.00E-04	0.11
D-Mannose-6-phosphate	2.88	1.00E-06	0.03
D-Ribulose 5-phosphate	3.48	5.00E-06	0.1
Deoxyguanosine diphosphate (dGDP)	1.32	1.11E-02	0.48
Dihydroxyacetone phosphate	1.24	1.22E-02	0.46

Note: *indicates that the metabolites with differential abundances between SZ3 and WT are detected in both the positive ionization mode (ESI+) and the ionization negative mode (ESI-), the one with less change folds is shown here. VIP represents variable importance for the projection, the VIP score is based on the variable weight value obtained by the OPLS-DA model to measure the influence intensity and interpretation ability of the expression pattern of each metabolite on the classification and discrimination of each group of samples and assists the screening of metabolites. VIP score>1 is used as the screening criterion. The *p*-value is the statistical analysis value of the two-tailed unpaired Student's *t*-test statistics of the metabolites in the pairwise comparison group. *p*-value<0.05 represents a significant difference metabolite, and 0.05<*p*-value<1 represents a different metabolite. FC: fold change.
