# Supplementary material for: Unlocking the potentials of cyanobacterial photosynthesis for directly converting carbon dioxide into glucose
Source: Nat Commun. 2023 Jun 9;14:3425. doi: 10.1038/s41467-023-39222-w (PMC10256809; doi:10.1038/s41467-023-39222-w)
Supplement: Supplementary file 3 — Description of Additional Supplementary Files [file 41467_2023_39222_MOESM3_ESM.pdf]

Description of Additional Supplementary Files

File Name: **Supplementary Data 1**
Description: Differential genes of SZ182 strain compared with SZ181 strain-RPKM.

File Name: **Supplementary Data 2**
Description: Differential genes of SZ3 strain compared with WT strain-RPKM.

File Name: **Supplementary Data 3**
Description: Metabolites with differentiated abundances between SZ3 and WT-NEG.

File Name: **Supplementary Data 4**
Description: Metabolites with differentiated abundances between SZ3 and WT-POS.

File Name: **Supplementary Data 5**
Description: Glk distributions.

File Name: **Supplementary Data 6**
Description: Primers and plasmids used in this study.

File Name: **Supplementary Data 7**
Description: Strains constructed in this study.
