# Supplementary material for: Unlocking the potentials of cyanobacterial photosynthesis for directly converting carbon dioxide into glucose
Source: Nat Commun. 2023 Jun 9;14:3425. doi: 10.1038/s41467-023-39222-w (PMC10256809; doi:10.1038/s41467-023-39222-w)
Supplement: Supplementary file 11 — Reporting Summary [file 41467_2023_39222_MOESM11_ESM.pdf]

nature portfolio

Corresponding author(s): Xuefeng Lu

Last updated by author(s): May 27, 2023

Reporting Summary

Nature Portfolio wishes to improve the reproducibility of the work that we publish. This form provides structure for consistency and transparency in reporting. For further information on Nature Portfolio policies, see our Editorial Policies and the Editorial Policy Checklist.

Statistics

For all statistical analyses, confirm that the following items are present in the figure legend, table legend, main text, or Methods section.

n/a	Confirmed	
☐	☒	The exact sample size (n) for each experimental group/condition, given as a discrete number and unit of measurement
☐	☒	A statement on whether measurements were taken from distinct samples or whether the same sample was measured repeatedly
☐	☒	The statistical test(s) used AND whether they are one- or two-sided<br>*Only common tests should be described solely by name; describe more complex techniques in the Methods section.*
☒	☐	A description of all covariates tested
☒	☐	A description of any assumptions or corrections, such as tests of normality and adjustment for multiple comparisons
☐	☒	A full description of the statistical parameters including central tendency (e.g. means) or other basic estimates (e.g. regression coefficient) AND variation (e.g. standard deviation) or associated estimates of uncertainty (e.g. confidence intervals)
☐	☒	For null hypothesis testing, the test statistic (e.g. F, t, r) with confidence intervals, effect sizes, degrees of freedom and P value noted<br>*Give P values as exact values whenever suitable.*
☒	☐	For Bayesian analysis, information on the choice of priors and Markov chain Monte Carlo settings
☒	☐	For hierarchical and complex designs, identification of the appropriate level for tests and full reporting of outcomes
☒	☐	Estimates of effect sizes (e.g. Cohen's d, Pearson's r), indicating how they were calculated

Our web collection on statistics for biologists contains articles on many of the points above.

Software and code

Policy information about availability of computer code

Data collection	1. The raw LC-MS data was converted by the ProteoWizard software (version 3.0.21229)<br>2. LightCycler 480 software 1.5 was used for RT-qPCR.<br>3. YZQ-201A13 was used for the determination of photosynthetic O2 evolution and dark respiration.
Data analysis	1. For the whole-genome re-sequencing, the reference sequence (Synechococcus elongatus PCC 7942, FACHB-805) was obtained from GenBank for read mapping using the BWA software (V0.7.8). SNPs and structure variation (SV) between reference sequences and sample sequences were identified by SAMtools (v0.1.18) and BreakDancer (version 1.4.5).<br>2. Code used for Glucokinase sequences of cyanobacteria are available on GitHub at https://github.com/yudifeiluo/cyanobacteria (Zenodo https://doi.org/10.5281/zenodo.7972565).<br>3. Cyanobacterial genomic sequences and their annotation from the NCBI assembly database using the NCBI-datasets tools (version13). The genome database was screened using the hmmsearch tool of the Hmmer package (version 3.1b2).<br>4. The number of reads corresponding to each gene was calculated using FeatureCounts (2.0.0).<br>5. For RNA-seq analysis, The reference genome and gene model annotation files (Synechococcus elongatus PCC 7942 and FACHB-805) were obtained from GenBank for read mapping using Bowtie2 (v2.3.4.3).

For manuscripts utilizing custom algorithms or software that are central to the research but not yet described in published literature, software must be made available to editors and reviewers. We strongly encourage code deposition in a community repository (e.g. GitHub). See the Nature Portfolio guidelines for submitting code & software for further information.

Data

Policy information about availability of data

All manuscripts must include a data availability statement. This statement should provide the following information, where applicable:

- Accession codes, unique identifiers, or web links for publicly available datasets
- A description of any restrictions on data availability
- For clinical datasets or third party data, please ensure that the statement adheres to our policy

Source data are provided with this paper in Source Data file. The accession number for the whole genome sequencing and transcriptome sequencing datasets reported in this paper is NCBI BioProject: PRJNA740138 (https://www.ncbi.nlm.nih.gov/bioproject/PRJNA740138). All raw data files for the untargeted metabolomics analysis have been deposited into CNGB Sequence Archive (CNSA) of China National GeneBank DataBase (CNGBdb) with accession number CNP0004406 (https://db.cngb.org/search/project/CNP0004406/). The identified metabolites were searched against the Kyoto Encyclopedia of Genes and Genomes database (https://www.kegg.jp/kegg/kegg2.html). Compound identification of metabolites by MS/MS spectra with an in-house database established with available authentic standards. The reference genome and gene model annotation files (Synechococcus elongatus PCC 7942 and FACHB-805) for whole genome re-sequencing and RNA-seq were obtained from GenBank [https://ftp.ncbi.nlm.nih.gov/genomes/all/GCA/000/012/525/GCA_000012525.1_ASM1252v1/]. All analytical data generated for the present study are available upon requests to the corresponding authors.

Human research participants

Policy information about studies involving human research participants and Sex and Gender in Research.

Reporting on sex and gender	N/A
Population characteristics	N/A
Recruitment	N/A
Ethics oversight	N/A

Note that full information on the approval of the study protocol must also be provided in the manuscript.

Field-specific reporting

Please select the one below that is the best fit for your research. If you are not sure, read the appropriate sections before making your selection.

☒ Life sciences ☐ Behavioural & social sciences ☐ Ecological, evolutionary & environmental sciences

For a reference copy of the document with all sections, see nature.com/documents/nr-reporting-summary-flat.pdf

Life sciences study design

All studies must disclose on these points even when the disclosure is negative.

Sample size	No statistical method was used to determine sample size. Sample size was chosen based on previous experience and standards in the field. The sample size has been shown in each figure legend and methods, and at least three biological replicates were used.
Data exclusions	No data was excluded from the manuscript.
Replication	All experiments were conducted more than twice. All attempts at replication were successful.
Randomization	The submitted work does not require randomization because our study did not involve assigning subjects into experimental test groups.
Blinding	The group allocation was not involved in our study. So investigators were not blind to the group allocation. Investigators were not blind during acquisition and analysis as well. Because the collected data was the result of quantitative data detected by instruments and was generally not affected by subjective factors.

Reporting for specific materials, systems and methods

We require information from authors about some types of materials, experimental systems and methods used in many studies. Here, indicate whether each material, system or method listed is relevant to your study. If you are not sure if a list item applies to your research, read the appropriate section before selecting a response.

Materials & experimental systems

n/a	Involved in the study
☒	☐ Antibodies
☒	☐ Eukaryotic cell lines
☒	☐ Palaeontology and archaeology
☒	☐ Animals and other organisms
☒	☐ Clinical data
☒	☐ Dual use research of concern

Methods

n/a	Involved in the study
☒	☐ ChIP-seq
☒	☐ Flow cytometry
☒	☐ MRI-based neuroimaging
